# Proteomic analysis of phosphoproteins sensitive to a phosphatidylinositol 3-kinase inhibitor, ZSTK474, by using SELDI-TOF MS

**DOI:** 10.1186/1477-5956-7-14

**Published:** 2009-03-31

**Authors:** Tetsuyuki Akashi, Takao Yamori

**Affiliations:** 1 Division of Molecular Pharmacology, Cancer Chemotherapy Center, Japanese Foundation for Cancer Research, Tokyo, Japan

## Abstract

**Background:**

Phosphoproteins play important roles in a vast series of biological processes. Recent proteomic technologies offer the comprehensive analyses of phosphoproteins. Recently, we demonstrated that surface-enhanced laser desorption/ionization time of flight mass (SELDI-TOF MS) would detect phosphoproteins quantitatively, which was a new application of SELDI-TOF MS.

**Results:**

We combined immobilized metal affinity chromatography (IMAC) with SELDI-TOF MS. After SELDI-TOF MS analysis of IMAC-enrichment phosphoproteins from A549 cancer cells, a series of protein peaks at 12.9, 12.8, 12.7 and 12.6 kDa was obtained in a mass spectrum. The peak intensities of these proteins decreased after a phosphatase treatment and, interestingly, they also decreased when the cells were pre-treated with a novel phosphatidylinositol 3-kinase (PI3K) inhibitor, ZSTK474, suggesting that these proteins were ZSTK474-sensitive phosphoproteins. Identity of the phosphoproteins, which were predicted as the multi-phosphorylated forms of 4E-binding protein 1 (4E-BP1) with the aid of TagIdent algorithm, was confirmed by immunoprecipitation and subsequent SELDI-TOF MS analysis. 4E-BP1 is a downstream component of the PI3K/Akt/mTOR pathway and it regulates protein synthesis. We also investigated the effect of ZSTK474 on 4E-BP1 phosphorylation using phospho-specific antibodies. ZSTK474, which have little inhibitory activity for mTOR, inhibited phosphorylation of Ser65, Thr70 and Thr37/46 in 4E-BP1. In contrast, rapamycin, an inhibitor of mTOR, blocked phosphorylation only of Ser65 and Thr70. These results suggest that ZSTK474 and rapamycin inhibited the phosphorylation of 4E-BP1 in a different manner.

**Conclusion:**

We identified a group of ZSTK474-sensitive phosphoproteins as the multi-phosphorylated form of 4E-BP1 by combining IMAC, SELDI-TOF MS and antibodies.

## Background

Phosphoproteins are involved in numerous signaling pathways in living cells. Recent development of proteomics-based technologies have provided many tools for analyzing phosphoproteins comprehensively [1-5]. One such technology is surface-enhanced laser desorption-ionization time-of-flight mass spectrometry (SELDI-TOF MS). Recently, we and another group successfully used SELDI-TOF MS to identify phosphoproteins in the crude cell extracts [6,7]. We further demonstrated that SELDI-TOF MS could quantitatively detect phosphoproteins [8] and identify phosphorylated sites of a phosphoprotein [8].

Class I phosphatidylinositol 3-kinase (PI3K), a heterodimer comprised of the regulatory and catalytic subunits, is a major signaling component downstream of receptor tyrosine kinases. The PI3K signaling pathway is frequently activated in various types of cancer cells, and is believed to promote cell proliferation, growth, and survival [9]. Mutations and/or amplification of PI3K gene have been found in human cancer cells derived from several tissues [9,10]. Thus, PI3K is a potential target for the development of drugs for cancer chemotherapy.

Although there are a number of PI3K inhibitors under development [11-16], as of yet none of them has been launched as an anticancer drug for clinical use. Recently, we developed a potent novel PI3K inhibitor, ZSTK474 [11], which competitively blocked the binding of ATP to the catalytic subunit of all class I PI3K isoforms [17], but did not significantly inhibit the activity of 140 other protein kinases including that of mTOR [11,17]. Furthermore, ZSTK474 inhibited several human cancer xenografts without severe toxicity [11]. Since it will be an advantage to know whether a potential drug is suitable for clinical use, a detailed knowledge of the molecular pharmacology of ZSTK474 is necessary to achieve this goal.

The aim of this study was to identify phosphoproteins whose phosphorylations were sensitive to ZSTK474 by combining immobilized metal affinity chromatography (IMAC) with SELDI-TOF MS and to investigate the effect of ZSTK474 on the phosphorylations in human cancer cells. In the present study, we identified a group of ZSTK474-sensitive phosphoproteins as the multi-phosphorylated form of 4E-binding protein 1 (4E-BP1).

## Results and discussion

### Analysis of phosphoprotein profiles by SELDI-TOF MS

**Figure 1:**
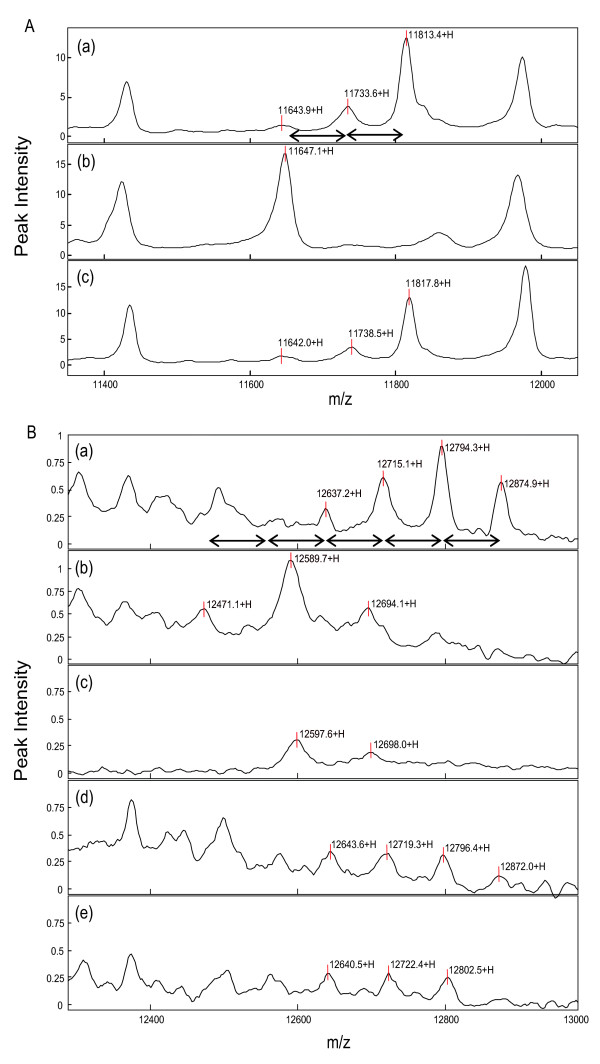
**Analysis of phosphoprotein profiles by SELDI-TOF MS**. Lysate from A549 cells was purified by IMAC resins and subsequently analyzed by SELDI-TOF MS as described in Methods. Two regions of SELDI-TOF MS spectra were shown (A and B). The eluate from the IMAC resin was treated without (A-a, B-a) or with (A-b, B-b) λ-PPase for 1 hr at 30°C, and then was applied onto the Q10 chips. The mass spectrum of the sole λ-PPase protein was also analyzed by SELDI-TOF MS (B-c). The A549 cells pre-treated with 0.5 μM (B-d) and 1.5 μM (A-c, B-e) of ZSTK474 for 30 min were lysed and processed for analysis by SELDI-TOF MS. The intensity of the 12.9 kDa protein peak decreased by ZSTK474 treatment in a dose dependent manner (B-a, d, and e). The left-right arrow indicates a mass difference of 80 Da that accounts for the MW of a phosphate group.

In order to efficiently capture and detect phosphoproteins by SELDI-TOF MS, we first purified and concentrated phosphoproteins from the lysate of A549 human lung cancer cells using IMAC resins. The eluted proteins from the resins were applied onto a strong anion-exchange array (Q10 chip) and subsequently analyzed by SELDI-TOF MS. As a result of this analysis, we also obtained several multi-peak containing protein profiles (data not shown). Since the mass of a phosphate group is 80 Da, dephosphorylation by a phosphatase would be expected to shift the peak of a phosphoprotein by a mass of 80 Da × *n *(where *n *is the number of phosphorylated sites) less than the original peak. Previously, we identified the phosphorylated form of the ribosomal P2 protein from crude extracts by using the SELDI-TOF MS technique [6]. In the present study also we obtained similar result (Fig. 1A). The single peak at 11.6 kDa in Fig. 1A-b is the non-phosphorylated form of ribosomal P2 according to our previous analysis. The 11.7 and 11.8 kDa proteins in Fig. 1A-a are the mono- and di- phosphorylated ribosomal P2, respectively, as the two protein peaks converged into a single peak after the λ-PPase treatment in Fig. 1A-b. Similarly, we detected several phosphoprotein candidates whose peaks disappeared when treated with λ-PPase (data not shown). We analyzed only intact proteins that were not treated with proteases. It might be possible that many phosphoproteins did not fall in the detectable range of SELDI-TOF MS in the present conditions. Among those phosphoprotein candidates, a series of 12.9, 12.8, 12.7 and 12.6 kDa proteins might be multi-phosphorylated forms of a protein, since the mass difference between each adjacent protein peak was 80 Da (Fig. 1B-a). Furthermore, λ-PPase treatment decreased these peak intensities and concomitantly increased the peak intensity at 12.5 kDa (12,471 Da in Fig. 1B-b) which was about 400 Da (80 Da × 5) smaller than the 12.9 kDa (12,875 Da in Fig. 1B-a), suggesting that the 12.5 kDa (12,471 Da in Fig. 1B-b) and 12.9 kDa (12,875 Da in Fig. 1B-a) proteins were the original one containing non-phosphorylated 5 phosphorylation sites and its penta-phosphorylated form, respectively. The peaks at 12.6 kDa (12,590 Da) and 12.7 kDa (12,694 Da) in Fig. 1B-b turn out to be derived from the λ-PPase, because the corresponding peaks (12,598 and 12,698 Da in Fig. 1B-c) were observed when λ-PPase alone was analyzed on the chip.

### Screening of ZSTK474-sensitive phosphoproteins

Among the candidate phosphoproteins, we next searched for the ZSTK474-sensitive phosphoproteins. When proteins were purified by IMAC from the ZSTK474-treated A549 cell extract and subsequently analyzed by SELDI-TOF MS, we found that the intensity of the 12.9 kDa protein peak was remarkably reduced in a dose dependent manner (Figs. 1B-d, e). In addition, the peak intensities of 12.8 and 12.7 kDa proteins also obviously decreased (Figs. 1B-d, e). These results implied that these proteins were from a ZSTK474-sensitive phosphoprotein that contained multiple phosphorylation sites. The peaks of ribosomal P2 protein (Fig. 1A-c) and the other phosphoprotein candidates (data not shown), however, remained unchanged upon ZSTK474 treatment, suggesting that the phosphorylation of these proteins were not affected by ZSTK474.

### Identification of the ZSTK474-sensitive phosphoproteins

We next used the TagIdent tool , which searches for candidate proteins from the Swiss-Prot database based on the input isoelectric point (p*I*) and molecular weight (MW), to predict the phosphoprotein candidates. Since the series of phosphoproteins was detected on the strong anion-exchange arrays (Q10 chip), we roughly estimated the p*I *of the targets within the acidic pH region, that is from 3.0 to 7.0. Then, we inputted the hypothetic MW values that were 80 Da × *n *smaller than the 12.9 kDa (12,875 Da in Fig. 1B-a), and searched the protein lists to find candidate phosphoproteins. When a p*I *of 5.0 (± 2.0) and a molecular mass of 12,475 Da (± 0.5%) were inputted in the TagIdent, 30 proteins were listed in the specified p*I*/MW range. Among these proteins, we noticed 4E-BP1 (accession no. Q13541) and considered it as our target phosphoproteins, because the Swiss-Prot suggested that it could be phosphorylated at multiple sites and also because it was known to be a downstream component of the PI3K/Akt/mTOR pathway [18].

**Figure 2:**
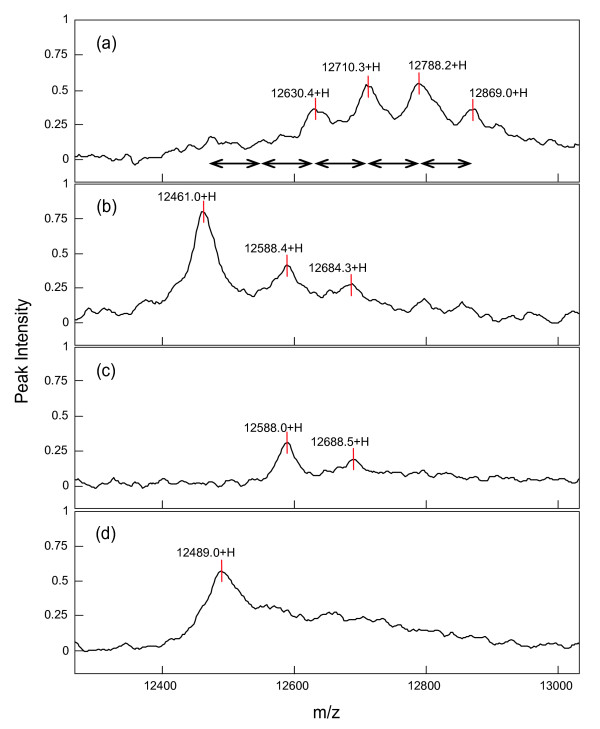
**Immunoprecipitation and subsequent SELDI-TOF MS analysis of the target proteins**. Extracts prepared from the A549 cells overexpressing 4E-BP1 were first incubated with anti-total-4E-BP1 antibody and then with protein G agarose. The bound proteins were eluted from the resins as described in Methods. Eluate from the untreated cells (a), eluate treated with λ-PPase for 1 hr at 30°C (b), and λ-PPase alone (c) were applied to NP20 chips and analyzed by SELDI-TOF MS. Lysate from the cells pre-treated with 1.5 μM ZSTK474 for 30 min was immunoprecipitated and processed for analysis by SELDI-TOF MS (d). The left-right arrow indicates a mass difference of 80 Da.

To confirm that the series of phosphoproteins is indeed 4E-BP1, we immunoprecipitated 4E-BP1 and its phosphorylated forms from the extract of A549 cells transiently overexpressing 4E-BP1 by using an anti-total-4E-BP1 antibody, and analyzed the immunoprecipitated proteins by SELDI-TOF MS after loading the immunoprecipitate onto a NP20 chip. As shown in Fig. 2a, a 12.9 kDa protein peak and three other peaks, each differing from the 12.9 Da peak by a mass of about 80 Da × *n*, were detected in the mass spectra. When the immunoprecipitate was treated with λ-PPase and subsequently analyzed by SELDI-TOF MS, a single peak at 12.5 kDa, other than the peaks derived from the λ-PPase, was observed (Fig. 2b). The mass difference between the 12.9 and 12.5 kDa protein peaks was about 400 Da, which corresponds to the sum of five phosphate groups (80 Da × 5). These results suggested that the target protein was 4E-BP1, and the 12.9 kDa protein peak was the penta-phosphorylated form of 4E-BP1. Although a MS/MS analysis is needed to prove that a mass shift of 80 Da really indicate a phosphorylation site, our experiments with a phosphatase and antibodies make it very likely that the signals indeed correspond to the suggested phosphoprotein.

Furthermore, when the A549 cells were pre-treated with 1.5 μM ZSTK474 for 30 min, and the cell extract was then immunoprecipitated with the anti-total-4E-BP1 antibody and subsequently analyzed by SELDI-TOF MS, a single peak at 12.5 kDa was detected in the mass spectra of the immunoprecipitate (Fig. 2d), suggesting that phosphorylation of all sites in 4E-BP1 were inhibited by ZSTK474. It is known that the hypophosphorylated 4E-BP1 binds to the eukaryotic translation initiation factor 4E (eIF4E) that recognizes the cap structure present at the 5' end of mRNAs, resulting in inhibition of cap-dependent translation initiation [19]. Therefore, our results, as described above, suggest that ZSTK474 may cause a reduction in protein synthesis by inhibiting 4E-BP1 phosphorylation, which in turn might lead to the slowing down of cell growth/cell size [20] and cell cycle progression [21].

### Inhibitory effect of ZSTK474 on 4E-BP1 phosphorylation

**Figure 3:**
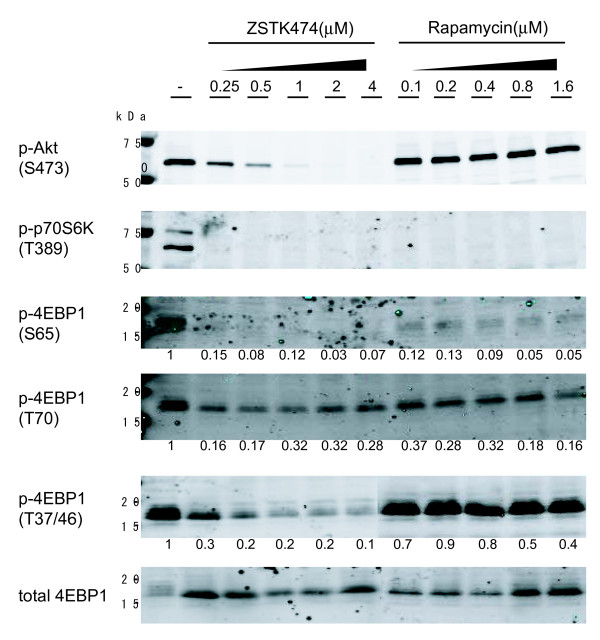
**Inhibitory effects of ZSTK474 on phosphoproteins**. Phosphorylation of Akt (Ser473), p70S6K (Thr389), 4E-BP1 (Ser65, Thr70, Thr37/46) and total 4E-BP1 were measured in 4E-BP1 over-expressing A549 cells by immunoblotting with respective antibodies. The cells were treated with indicated concentrations of ZSTK474 (0.25, 0.5, 1, 2, and 4 μM) or rapamycin (0.1, 0.2, 0.4, 0.8, and 1.6 μM) for 30 min. Intensity ratio of phosphorylated form/total form of 4E-BP1 was indicated under each lane. ZSTK474 inhibited phosphorylations of each downstream component of PI3K in a dose dependent manner. In contrast, rapamycin did not inhibit phosphorylation of Thr37/46 in 4E-BP1 as well as that of Ser473 in Akt.

The regulation of 4E-BP1 phosphorylation at each potential phosphorylation-site is not clearly understood. Therefore, we next analyzed the ZSTK474-mediated inhibition of phosphorylation in 4E-BP1 (Ser65, Thr70, and Thr37/46) and also in two other components in the PI3K pathway, namely Akt (Ser473) and p70 S6 kinase (p70S6K) (Thr389), using A549 cells overexpressing 4E-BP1 and immunoblotting with phospho-specific antibodies (Fig. 3). The PI3K inhibitor ZSTK474 inhibited phosphorylation of Ser473 in Akt, Thr389 in p70S6K, as well as phosphorylation of all sites in 4E-BP1 (Fig. 3). In the case of phosphorylation of Thr70 in p-4E-BP1, the lowest inhibitor concentrations reduced the phosphorylation degree significantly. However, no dose response was observed (Fig. 3). Its molecular mechanism remains to be clarified. On the other hand, the mTOR inhibitor rapamycin did not inhibit the phosphorylation of Thr37/46 in 4E-BP1 or that of Ser473 in Akt (Fig. 3), although it inhibited the phosphorylation of Thr389 in p70S6K, and Ser65 and Thr70 in 4E-BP1. These results suggest that ZSTK474 and rapamycin differentially inhibited the phosphorylation of 4E-BP1. It seems quite reasonable because we previously demonstrated that ZSTK474 did not inhibit mTOR [11,17]. Rapamycin reportedly blocks the formation of mTOR-rapter-4E-BP complex, which causes the inhibition of the phosphorylation of Ser65 and Thr70, but not the phosphorylation of Thr37/46 [22,23]. Unknown kinase(s) could be responsible for the phosphorylation of Thr37/46, and ZSTK474 might inhibit such unknown pathways. However, the mechanism by which ZSTK474 caused the inhibition of 4E-BP at all the four sites remains unknown. ZSTK474, which is a potent and narrow-spectrum PI3K inhibitor [11,17], might become an invaluable tool for analyzing the mechanism in the future.

## Conclusion

In this study, we developed an analytical method for identifying drug-sensitive phosphoproteins by combining IMAC, SELDI-TOF MS and antibodies. By using this method, we identified a series of phosphoproteins as the multi-phosphorylated 4E-BP1 proteins whose phosphorylation were inhibited by ZSTK474. This result suggested that our method is useful to identify drug-sensitive phosphoproteins. In addition, we identified the phosphorylation status of 4E-BP1 at its multiple phosphorylation sites by SELDI-TOF MS. These results together with our previous report [8] suggest that the SELDI-TOF MS technique is an useful tool for quantitatively analyzing phosphoproteins. This technique can be applicable for analyzing other post-translational modifications, such as phosphorylation, glycosylation, acetylation, etc.

## Methods

### Materials

ZSTK474 was provided by Zenyaku Kogyo Co. (Tokyo, Japan). Rapamycin was purchased from Sigma (St. Louis, MO, USA). Antibodies to phospho-Akt (Ser473) [25], phospho-p70S6K (Thr389) [25], phospho-4E-BP1 (Ser65) [24,25], phospho-4E-BP1 (Thr70) [24,25], phospho-4E-BP1 (Thr37/46) [25], and total-4E-BP1 [25] were purchased from Cell Signaling Technology (Beverly, MA, USA) [24,25]. The phospho-Akt (Ser473) and 4E-BP1 (Ser65) antibodies were monoclonal, and the others were polyclonal.

### Cell culture and drug treatment

Human lung cancer cell line, A549, was cultured in RPMI 1640 supplemented with 5% fetal bovine serum and kanamycin (10 μg/ml) at 37°C in humidified air containing 5% CO_2_. Cells were seeded at 5 × 10^6 ^– 6.8 × 10^6 ^cells in 100-mm dish and grown over night. Cells were first preincubated without or with the indicated concentration of a given inhibitor (ZSTK474 or rapamycin) for 30 min, followed by incubation with 10 ng/ml epidermal growth factor (EGF) for 10 min, and finally they were washed with cold PBS and frozen in liquid N_2_.

### IMAC

Phosphoproteins from cell lysate were purified with a PhosphoProtein Purification Kit (QIAGEN, Hilden, Germany) according to a modified version of the manufacturer's protocol. Briefly, frozen cells, collected from three 100-mm dishes for each treatment, were lysed with 1 ml of a lysis buffer (QIAGEN) containing protease inhibitors, benzonase and 0.5% (v/v) NP-40, and incubated for 30 min on ice. After centrifugation at 20,000 × g for 20 min, the protein content of the supernatant was estimated using a Protein Assay Kit (Bio-Rad Laboratories, Hercules, USA), and the supernatant was then diluted to a protein concentration of 0.1 mg/ml with the lysis buffer (QIAGEN) containing 0.25% (w/v) CHAPS. After performing chromatography on the IMAC column following manufacturer's instructions, the eluted fractions were desalted and concentrated by centrifugation at 20,000 × g using a Vivaspin 500 tube (Sartorius, Goettingen, Germany).

### SELDI-TOF MS

The SELDI-TOF MS analysis was performed as previously described [6,8]. Phosphoprotein profiles were obtained by using the strong anion-exchange arrays (Q10, Bio-Rad). Immunoprecipitated samples were applied to normal phase protein array (NP20, Bio-Rad). Each spot of the array was finally allowed to dry, subsequently two aliquots of 0.5 μl saturated sinapinic acid in 50% acetonitrile in water containing 0.5% trifluoroacetic acid were applied on each spot and air-dried.

### Phosphatase treatment

Lambda protein phosphatase (λ-PPase; New England Biolabs, MA, USA), which dephosphorylates all types of phosphorylated amino acid residues (i.e., p-Ser, p-Thr, and p-Tyr), was used for dephosphorylation of phosphoproteins as previously described [6].

### Cloning, Construction of Expression Plasmid, and Transfection

Human 4E-BP1 cDNA was cloned into the expression vector pcDNA3.1 (Invitrogen, Carlsbad, USA) to transiently overexpress the native 4E-BP1 protein in order to enhance the detection of phosphorylated 4E-BP1 by SELDI-TOF MS and immunoblotting. A549 cells were selected as hosts to express the recombinant protein, and were transfected with the 4E-BP1 expression plasmid using the Lipofectamine 2000 reagent (Invitrogen) as described previously [8].

### Immunoprecipitation

Cells expressing the recombinant protein were lysed in a lysis buffer (50 mM HEPES-NaOH, pH 7.5, 0.5% NP-40, 1 mM sodium orthovanadate, 25 mM sodium fluoride, 15 mM pyrophosphate, and 5 mM EDTA) containing protease inhibitors [0.1 mM PMSF, 1 μg/ml leupeptin, 1 μg/ml pepstatin] and incubated for 30 min on ice. The supernatant obtained after centrifugation at 20,000 × *g *for 20 min was diluted with the lysis buffer and incubated with the anti-total-4E-BP1 antibody for 16 h at 4°C, followed by incubation with the protein G agarose (Upstate, NY, USA) for 2 hr at 4°C with gentle shaking. The resin was subsequently washed three times with 10–15 resin volumes of lysis buffer and once with HEPES buffer (20 mM HEPES, pH 7.5, 50 mM NaCl). Proteins adsorbed to the resin were eluted by incubation with 5–10 resin volumes of 0.1 M glycine, pH 2.5 for 15 min on ice. The eluate was neutralized by the addition of appropriate amount of saturated Tris, and was desalted and concentrated by centrifugation at 20,000 × *g *using Vivaspin 500 tube.

### Immunoblot analysis

Immunoblotting was performed as previously described [11]. An appropriate Alexa Fluor 680-labeled anti-rabbit IgG (Invitrogen) was used as a secondary antibody, and immunoreactive protein bands were analyzed using the Odyssey infrared imaging system (Li-Cor, Lincoln, NE, USA).

## Abbreviations

SELDI-TOF MS: surface-enhanced laser desorption/ionization time of flight mass; PI3K: phosphatidylinositol 3-kinase; 4E-BP1: 4E-binding protein 1; λ-PPase: lambda protein phosphatase; p*I*: isoelectric point; MW: molecular weight

## Competing interests

The authors declare that they have no competing interests.

## Authors' contributions

TA designed and performed all experimental work and drafted the manuscript. TY is the chief of the laboratory, and he coordinated the study and revised the manuscript. All authors read and approved the final manuscript.
